# Disynaptic Subthalamic Input to the Posterior Cerebellum in Rat

**DOI:** 10.3389/fnana.2017.00013

**Published:** 2017-02-28

**Authors:** Saad Jwair, Patrice Coulon, Tom J. H. Ruigrok

**Affiliations:** ^1^Department of Neuroscience, Erasmus Medical CenterRotterdam, Netherlands; ^2^Institut de Neurosciences de la Timone, Aix-Marseille Université, CNRSMarseille, France

**Keywords:** rabies virus, cerebellar cortex, subthalamic nucleus, pontine nuclei, basal ganglia, pedunculotegmental nucleus

## Abstract

In the last decade, the interplay between basal ganglia and cerebellar functions has been increasingly advocated to explain their joint operation in both normal and pathological conditions. Yet, insight into the neuroanatomical basis of this interplay between both subcortical structures remains sparse and is mainly derived from work in primates. Here, in rodents, we have studied the existence of a potential disynaptic connection between the subthalamic nucleus (STN) and the cerebellar cortex as has been demonstrated earlier for the primate. A mixture of unmodified rabies virus (RABV: CVS 11) and cholera toxin B-subunit (CTb) was injected at places in the posterior cerebellar cortex of nine rats. The survival time was chosen to allow for disynaptic retrograde transneuronal infection of RABV. We examined the STN for neurons infected with RABV in all nine cases and related the results with the location of the RABV/CTb injection site, which ranged from the vermis of lobule VII, to the paravermis and hemispheres of the paramedian lobule and crus 2a. We found that cases with injection sites in the vermis of lobule VII showed prominent RABV labeling in the STN. In contrast, almost no subthalamic labeling was noted in cases with paravermal or hemispheral injection sites. We show circumstantial evidence that not only the pontine nuclei but also the pedunculotegmental nucleus may act as the intermediary in the connection from STN to cerebellar cortex. This finding implies that in the rat the STN links disynaptically to the vermal part of lobule VII of the cerebellar cortex, without any major involvement of the cerebellar areas that are linked to sensorimotor functions. As vermal lobule VII recently has been shown to process disynaptic input from the retrosplenial and orbitofrontal cortices, we hypothesize that in the rat the subthalamic input to cerebellar function might be used to influence more prominently non-motor functions of the cerebellum than motor functions. This latter aspect seems to contradict the primate results and could point to a more elaborate interaction between basal ganglia and cerebellum in more demanding motor tasks.

## Introduction

The basal ganglia and the cerebellum are major subcortical brain structures that control mainly motor, but also non-motor aspects of behavior initiated by the cerebral cortex (Gold et al., 1989; Middleton and Strick, 2000; Schmahmann and Caplan, 2006; Strick et al., 2009; D’Angelo and Casali, 2012; Schultz, 2016). Traditionally, the basal ganglia and the cerebellum were thought to function in parallel to each other, by having efferent projections to essentially non-overlapping regions of thalamic nuclei (Ilinsky and Kultas-Ilinsky, 1984; Deniau et al., 1992), thereby only interacting at cerebral cortex level (Percheron et al., 1996; Teune et al., 2000; Gallay et al., 2008). In the last decade, however, the use of transneuronal tracers has shown the existence of subcortical disynaptic connections between the basal ganglia and the cerebellum (Bostan et al., 2010). These connections suggest that the basal ganglia and the cerebellum, at least to some extent, might work in concert at a subcortical level.

After establishing a link from the cerebellar nuclei, by way of thalamic nuclei, to the striatum in macaque (Hoshi et al., 2005), Peter Strick’s group established a disynaptic subcortical link between the subthalamic nucleus (STN) and the cerebellum, in particular to lobule VII B and crus II of the cerebellar cortex (Bostan and Strick, 2010). Based on the differential topographically organized connections to both investigated cerebellar regions they postulated that this connection plays a role in both motor and non-motor functions of the cerebellum (Bostan and Strick, 2010; Bostan et al., 2010). Imaging studies that use diffusion magnetic resonance imaging and tractography have shown that this disynaptic connection between the STN and the cerebellar cortex may also be present in the human brain (Pelzer et al., 2013; Milardi et al., 2016). As such, these findings provide neuroanatomical evidence for the putative role of an affected interplay between basal ganglia and cerebellum in movement disorders. This has been largely derived from clinical and animal studies that showed a cerebellar component in disorders typically associated with basal ganglia dysfunction like Parkinson disease (especially in the generation of rest tremors; Benabid et al., 1991; Rascol et al., 1997; Wu and Hallett, 2005, 2013) or dystonia (Chen et al., 2014).

In rodents, a subcortical connection relating cerebellar output to basal ganglia functioning and its potential for inducing dystonia has recently been studied using wildtype and genetically modified mice (Fremont et al., 2015; Liu et al., 2015; also see Shakkottai et al., 2016). However, a reverse subcortical pathway from the basal ganglia to the cerebellum, to our knowledge, has not been demonstrated or investigated in rodents. Given the demonstration of this pathway in the primate and the surge of recent interest in the interplay between basal ganglia and cerebellum, we wanted to see if the STN—cerebellar connection could also be established in rodents and to what extent it differed with that of primates. Therefore, similar to the primate study by Bostan et al. (2010), we have studied a potential disynaptic connection between the STN and the cerebellar cortex in the rat by injecting a mixture of unmodified rabies virus (RABV: CVS 11) and cholera toxin B-subunit (CTb) at several places of the posterior cerebellar cortex (Suzuki et al., 2012). In total, nine small and confined injections were made in the posterior cerebellar cortex ranging from the vermis of lobule VII, to the paravermis and hemispheres of the paramedian lobule and crus IIb. The survival time was set to allow for disynaptic retrograde transneuronal infection of RABV, the resulting second order labeling was then related to the RABV/CTb injection site.

The results indicate that in the rat a disynaptic connection is present but more prominently reaches the vermis as compared to the hemispheres of lobule VII or crus2b.

## Materials and Methods

The experiments with RABV/CTb injections were all performed on male Wistar rats (200–300 g), which have also been used previously in a related but different study (Suzuki et al., 2012). Surgical procedures adhered to the European guidelines for the care and use of laboratory animals and were approved by the ethics committee in Neurosciences at the Institut de Neurosciences de la Timone, INT-Marseille. This study was carried out in accordance with the recommendations of the same committee. The protocol was approved by the same Ethics committee in Neurosciences (n° 02167–01). Vaccinated personnel conducted all RABV handling and surgery and animal care procedures were carried out at the appropriate biosafety containment level (level 2) in the lab of PC (Marseille). In all nine cases the unmodified “French” fixed strain of the Challenge Virus Standard (CVS) 11 was used as a tracer (Ugolini, 2011; Ruigrok et al., 2016).

### Surgical Procedures and Injections

As the actual injections have been described earlier (Suzuki et al., 2012), we will only recapitulate the main procedures briefly. The animals, after inducing ketamine/xylazine anesthesia, were placed in a stereotactic head holder (David Kopf Instruments). The vermis and the right side of the posterior cerebellum were exposed by incision of the skin overlying the occipital bone and the neck, separation of the neck muscles and partial removal of the occipital bone. After making a small incision in the dura, a fine micromanipulator driven Hamilton needle was horizontally and under visual guidance, introduced into either the vermis of lobule VII (*n* = 3), paramedian lobule (*n* = 4) or crus 2b (*n* = 2). The Hamilton needle, by way of thin plastic tubing, filled with water, was connected to a motorized Hamilton syringe. A small air bubble was introduced between the injectate and the rest of the water-filled tube and was used to monitor the injection. A volume of 150 or 200 nL of a mixture of 1 part 1% CTb (low salt; List Biological Laboratories, 1% w/v in 0.2 M phosphate buffer (PB), pH 7.4) and 4 parts RABV (Ruigrok et al., 2008) was injected in every case at a depth of 300–600 μm below the surface. All animals but one (survival time of case 1080 was 50 h) were allowed to survive for 48 h, during this period no behavioral changes of the animals were noted.

### Histology

After a survival time of 48 h the animals, under deep sodium pentobarbitone anesthesia, were flushed through the heart with saline and infused with 4% paraformaldehyde in PB. The brains were extracted, postfixed, embedded in gelatin and finally sectioned transversally at 40 μm with a freezing microtome. Sections were collected serially in eight vials for each case such that each vial contained a complete one out of eight series of sections. We used two vials (1 and 5) for RABV immunohistochemistry as well as two vials (2 and 6) for CTb immunolabeling. RABV immunohistochemistry was performed on free-floating sections using anti-rabies phosphoprotein mouse monoclonal antibody (dilution 1:5000) for 48–72 h. This antibody (31G10) was isolated in the Institut de Biologie Intégrative de la Cellule at Gif-sur-Yvette, France (Raux et al., 1997) and has been used successfully in many earlier publications (Ruigrok et al., 2008, 2016; Salin et al., 2008; Coulon et al., 2011; Deng et al., 2015; Kasumacic et al., 2015), then rinsed and incubated with rabbit anti-mouse horseradish peroxidase followed by 3,3diaminobenzidine-tetrahydrochloride (DAB) and peroxide solution for visualization. The sections in jar 2 and 6 were incubated in a goat anti-CTb antibody (List Biological Laboratories, dilution 1:15000), rinsed and incubated in biotinylated rabbit anti-goat that reacted with avidin-biotin complex followed by DAB+ for visualization (Suzuki et al., 2012). Finally, all sections of each jar were serially mounted, thionin counterstained and coverslipped with Permount.

For this study, sections from two male Wistar rats incubated with a monoclonal antibody against choline acetyltransferase (ChAT) were re-examined. The monoclonal antibody was a kind gift of Dr. M.R. Cozzari (Institute for Cell Biology, Rome, Italy) to Dr. D. Jaarsma (Department of Neuroscience, Erasmus MC Rotterdam, Netherlands) and specifics on the production and specificity of the antibody were detailed by Jaarsma et al. (1996). The sections used were from experiments that were part of a study on the origin of cholinergic innervation of the cerebellum by Jaarsma et al. (1997). Briefly, the 40 μm transverse sections were incubated with the monoclonal ChAT antibody (1:1000), a biotinylated secondary mouse antibody (1:200), Avidin-biotin-peroxidase complex (Vector laboratories) and DAB visualization.

### Analysis

Sections were examined with a Leica DMR microscope equipped with a digital camera (Leica DFC-450). Photopanels were constructed with Adobe Photoshop and Illustrator CS6. Injection sites were evaluated using the CTb series of sections and its size and location was entered on a surface rendering of caudal cerebellum (Suzuki et al., 2012). In addition, we used the CTb labeling in the inferior olive to determine the actual spread of the injection by relating this to the parasagittal zones of the cerebellar cortex described by Voogd et al. (2003) that all receive a distinct input from a subset of the inferior olivary nuclei (Voogd and Ruigrok, 2004; Pijpers et al., 2006). Details of the presently examined injections are described in Suzuki et al. (2012).

Plots of all RABV-labeled neurons in serial sections (1 out of 4 series, i.e., at 160 μm intervals) at the level of the STN were made using a motorized Olympus BH microscope equipped with a Lucivid miniature monitor and Neurolucida^TM^ software (Microbrightfield). Different symbols were used for RABV-labeled neurons within and outside of the confines of the STN. However, the present study only reports on RABV-labeled cells within the STN. Color-coded plots of the distribution of STN labeled neurons were made using standard Matlab (Mathworks) routines (Suzuki et al., 2012) and is further explained in Figure 2. Graphs were constructed with Excel^TM^ (Microsoft Office 2010).

## Results

**Figure 1:**
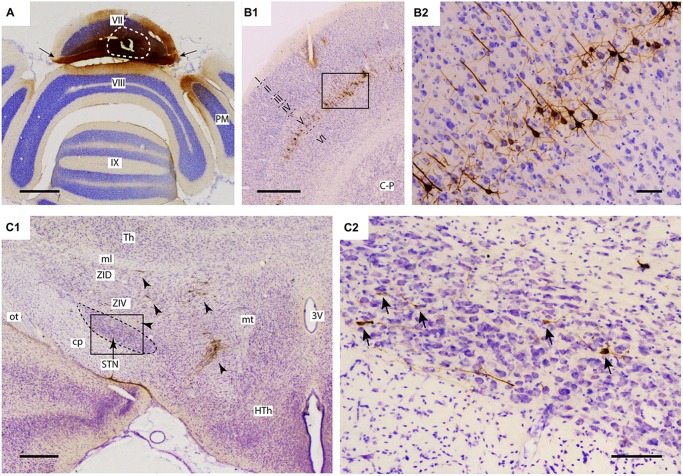
**Injection of rabies virus (RABV)/cholera toxin B-subunit (CTb) mixture results in 2nd order labeling of pyramidal cells in the cerebral cortex and of neurons in the STN. (A)** Injection site in the vermis of lobule VII as visualized by CTb immunohistochemistry. The white circle indicates the injection site. Labeling in the molecular layer indicates labeled parallel fiber bundles (arrows). **(B1,B2)** Overview and detail of RABV infection in the cerebral cortex, where only pyramidal neurons in layer V were labeled. **(C1,C2)** Overview and detail of RABV-infected neurons in ZI and lateral HTh (arrowheads in **(C1)** and STN (arrows in **(C2)**). Abbreviations: 3V, third ventricle; cp, cerebral peduncle; C-P, caudate-putamen; HTh, hypothalamus; ml, medial lemniscus; mt, mammillothalamic tract; ot, optic tract; PM, paramedian lobule; STN, subthalamic nucleus; Th, thalamus; ZID, dorsal zona incerta; ZIV, ventral zona incerta. Scale bars equal 1000 μm in **(A,B1)**, 500 μm in **(C1)** and 100 μm in **(B2,C2)**.

A total of nine injections with a mixture of RABV and CTb into lobule VII were evaluated for the present study. Three injections were confined to the vermis, four injections to the paravermal and hemispheral parts of the paramedian lobule and the remaining two injections were centered on crus 2B. The location and size of the injection was evaluated in CTb-processed sections (Figure 1A) and, as the organization of the olivocerebellar projection is particularly well known in rats (Sugihara and Shinoda, 2004; Voogd and Ruigrok, 2004; Pijpers et al., 2005), incorporated evaluation of the extent of retrograde labeling in the inferior olive (Suzuki et al., 2012). Retrograde, i.e., somatic, labeling of neurons outside of the cerebellum was carefully evaluated in both CTb- and RABV-processed sections.

For proper evaluation of the results it is vital to demonstrate unequivocally that no transneuronal labeling beyond second-order could have occurred, as this would dramatically increase potential routes of infection. The restriction of transneuronal labeling to one synaptic step was confirmed in the cerebral cortex, where in all studied cases, RABV -infected neurons were always confined to layer V and resembled pyramidal neurons (Bostan and Strick, 2010). No RABV labeling was encountered in other cortical layers (Figures 1B1,B2) or in the dorsal thalamus (not shown). As the pontine nuclei as well as the reticulotegmental nucleus of the pons both serve as major sources of mossy fibers to lobule VII and receive their major afferent input from layer V pyramidal cells of the cerebral cortex (e.g., see Ruigrok et al., 2015), this observation was in line with the disynaptic corticopontocerebellar pathway. Moreover, this indicated that all other RABV labeling was due to either first- or second-order infection.

In the diencephalon, although no RABV labeling was encountered in the dorsal thalamus and epithalamus, many infected neurons were seen in the subthalamic region, in particular in the prerubral field, fields of Forel, zona incerta and, more sparsely, within the STN. As these areas did not contain any CTb-labeled neurons, we conclude that the RABV labeling consisted of second-order infected neurons only. Scattered RABV labeling was also observed in hypothalamic areas, where, occasionally, also a CTb-labeled neuron could be observed (Dietrichs and Haines, 1989). Here, however, we will further focus on RABV labeling observed within the STN (Figures 1C1,C2). In evaluating this labeling it is also important to note that no RABV infected neurons could be observed in the external globus pallidus.

**Figure 2:**
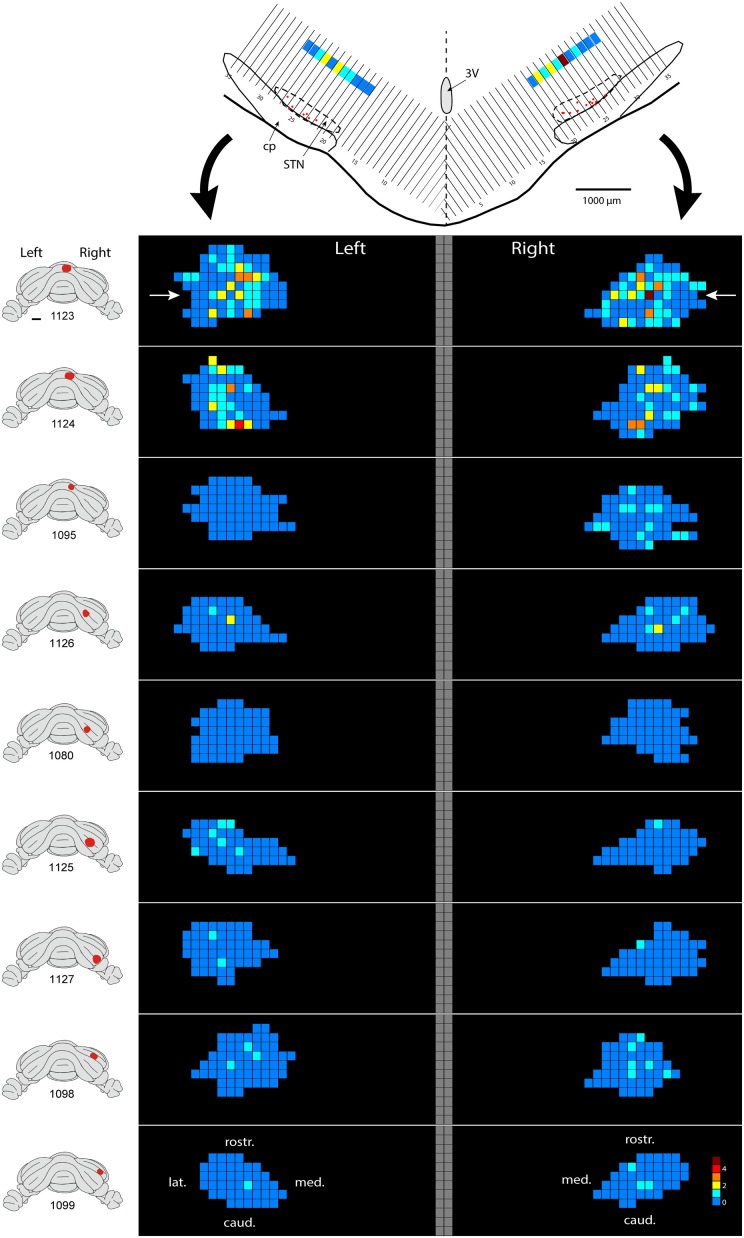
**Density plot of RABV-infected neurons in the STN.** This figure shows the results of nine cases with RABV/CTb injections in the posterior cerebellum and with resultant second order RABV labeling in the STN. The STN of the left and right side is shown in a dorsal view. The STN is binned in 160 μm-wide sections according to the top figure and number of labeled cells within a bin is color-coded. The “angled” left and right hand bins are rotated to a horizontal plane (black arrows). The position of the transverse section depicted at the top is indicated with white arrows in the top density figure (case 1123). A diagram of the location of the injection site is depicted on standardized caudal views of the rat cerebellum at the left hand side of the density figures.

**Figure 3:**
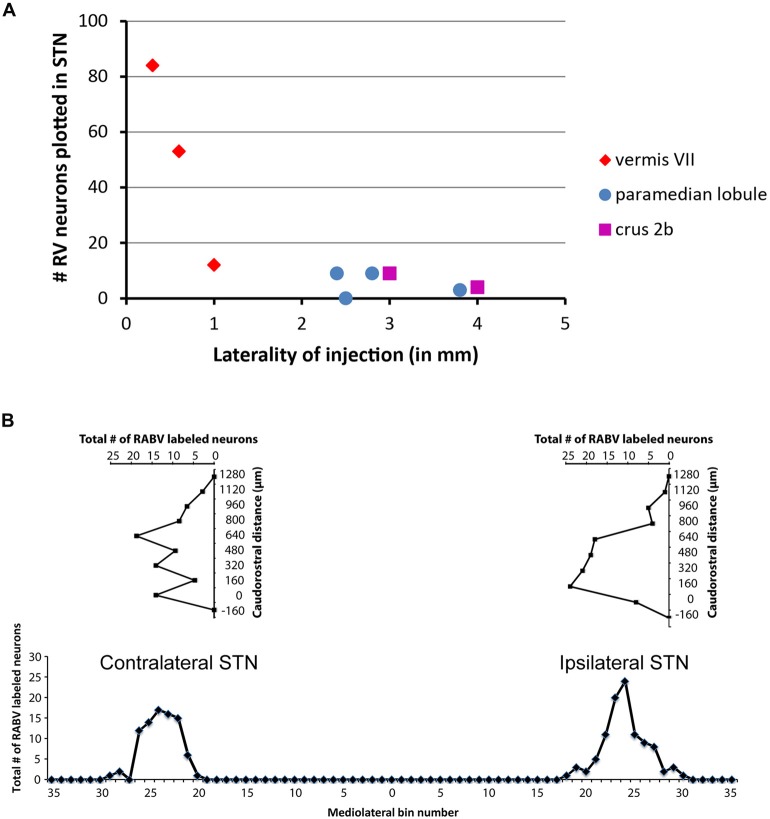
**Number of RABV-infected neurons in STN is related to laterality of injection site but shows no specific distribution. (A)** Diagram depicting the laterality of the center of the injection site and the resulting total count of RABV-infected neurons in the STN in a series of one in four examined sections of all examined cases (*n* = 9). **(B)** Diagrams indicating the summed distribution of RABV-infected neurons within the STN of all examined cases. Top diagrams depict the rostrocaudal distribution as based on the summed total of RABV neurons per section (horizontal axis) setting the first section with STN from caudal at 0 (vertical axis). Bottom diagram shows the mediolateral distribution based on the summed totals of labeled neurons per bin (also see Figure 2).

Only in one of the examined cases (case 1080), not a single RABV-infected neuron was observed within the STN despite infection of neurons in the zona incerta and prerubral field. The remaining eight cases all showed at least several and up to 84 infected STN neurons (determined in a one out of four series of plotted sections). Figure 2 shows the location and density of RABV-labeled neurons in color-coded diagrams based on 160 μm wide bins that were oriented along the long axis of the STN. The binning diagram is seen at the top of Figure 2. After color-coding the number of RABV neurons within every bin overlying the STN bin and flattening the result, a dorsomedial view of both the ipsi- (right side) and contralateral (left side) of the STN is presented. The accompanying injection site is indicated in a figurine of the rat cerebellum as seen from caudal. It is striking that the number of RABV-labeled neurons in the STN was most impressive when the injection was centered on the vermis of lobule VII (top two cases). Injections in the paramedian lobule or crus 2A resulted in only a few scattered RABV-infected neurons that were rather evenly distributed over the contra- and ipsilateral STN (Figure 3A). Figure 3B shows the summed mediolateral and rostrocaudal distribution of STN labeling of all eight cases with RABV neurons in the STN. These diagrams show that these neurons could be found throughout every aspect of the STN but mostly in the central part, which seems to correlate well with the general distribution of the number of STN neurons in this lens-shaped nucleus.

**Figure 4:**
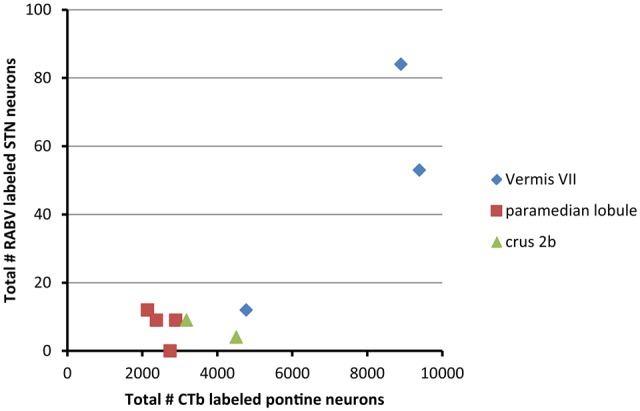
**RABV labeling in STN correlates with high numbers of CTb-labeled pontocerebellar neurons.** This diagram shows that the two cases with relatively high numbers of RABV-infected neurons in the STN also demonstrated large numbers of retrogradely CTb-labeled neurons in the pontine nuclei. No obvious correlations were observed between the numbers of STN and pontine neurons of the paramedian lobule cases or crus 2b cases. Numbers of CTb-labeled neurons in the basal pons are from Suzuki et al. (2012).

From these results it can be concluded that in the rat the vermis of lobule VII is more prominently reached by a disynaptic connection from the STN than its hemisphere. We have asked ourselves what the intermediary of this connection might be. It has been proposed that the pontine nuclei could be a likely candidate (Bostan and Strick, 2010). As, in the course of another study (Suzuki et al., 2012) we have plotted and determined the number of CTb-labeled neurons within the basal pontine nuclei and the reticulotegmental nucleus of the pons of the cases examined in the present study, we correlated the summed numbers of CTb-labeled pontine neurons with the numbers of plotted RABV neurons in the STN (Figure 4). It is obvious that both cases with vermal injections that resulted in the highest numbers of STN labeled neurons also harbored the highest number of CTb-labeled neurons in the pontine nuclei.

**Figure 5:**
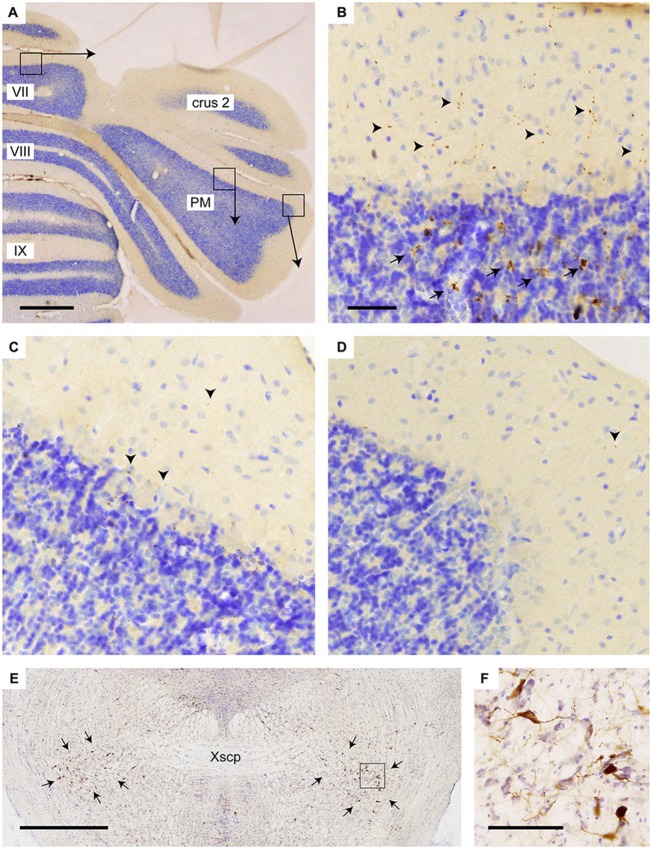
**Increased density of choline acetyltransferase (ChAT)-labeling in vermis of lobule VII as compared to PM. (A)** Overview of transverse section through the posterior cerebellum indicating three regions that are shown enlarged in **(B–D)**; **(B)** varicose (arrowheads) and mossy fiber rosette (arrows) ChAT labeling in the vermis; **(C)** only sparse punctate ChAT labeling (arrowheads) is observed around the Purkinje cells labeling in the central part of the PM; **(D)** Hardly any punctate labeling is observed in the lateral-most aspect of the PM. **(E)** Overview showing RABV labeling of neurons in the pedunculopontine tegmental nucleus (PPTg) area (indicated by arrows) in case 1124 (injection of vermis lobule VII). **(F)** Detail of boxed area in **(E)**. Abbreviation: PM, paramedian lobule; Xscp, decussation of the superior cerebellar peduncle. Scale bar equals 1 mm in **(A,E)** and 100 μm in **(B)**, the latter also for panels **(C,D)**.

**Figure 6:**
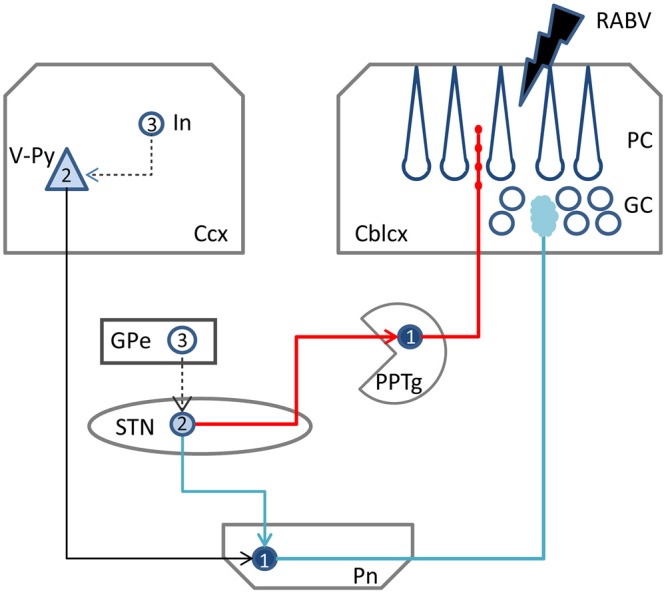
**Schematic diagram of the two potential routes from the STN to the cerebellar cortex.** RABV injected in the cerebellar cortex could be taken up by mossy fibers resulting in 1st order labeling in the Pn followed by subsequent 2nd order labeling in layer V of the Ccx and, potentially 2nd order labeling in the STN (blue route). Alternatively, ChAT varicose terminals may become infected resulting in 1st order labeling in PPTg and subsequently in 2nd order labeling in the STN (red route). RABV infected cortical interneurons and RABV neurons in the external globus pallidus, which would indicate 3rd order labeling, were never encountered and, therefore, are connected by a hatched line. Numbers in neurons indicated order of labeling. Abbreviations: Cblcx, cerebellar cortex; Ccx, cerebral cortex; GC, granular layer; GPe, external globus pallidus; In, cortical interneuron; PC, Purkinje cell layer; Pn, pontine nuclei; PPTg, pedunculopontine tegmental nucleus; STN, subthalamic nucleus; V-Py, pyramidal cells in layer V.

Yet, we think alternative pathways should also be considered as, in rodents, an STN projection to the basal pontine nuclei has not been reported in an extensive retrograde study (Mihailoff et al., 1989) and also with anterograde tracing an STN projection to the pontine nuclei is not observed in mice (e.g., see case 146986331 image 74–75 for the STN injection site and images 89–97 (source: Website: © 2015 Allen Institute for Brain Science. Allen Mouse Brain Connectivity Atlas [Internet]. Available from: http://connectivity.brain-map.org). Therefore, we also want to propose a potential alternative route. The STN mostly is a source of afferents of the entopeduncular nucleus (i.e., internal part of the globus pallidus) and the substantia nigra. In addition, connections have been described to the pedunculopontine tegmental nucleus (PPTg; Takada et al., 1988; Granata and Kitai, 1989; Kita and Kitai, 1991; Semba and Fibiger, 1992). As the PPTg is known to contain many cholinergic neurons and, among many other targets, also projects to the cerebellum where efferents terminate as thin varicose fibers in the upper granular and deep molecular layer (Jaarsma et al., 1997), we have qualitatively examined the density of these cholinergic fibers in lobule VII, paramedian lobule and crus 2b. Figure 5 illustrates that, indeed, the density of varicose ChAT-positive fibers is highest in the vermis and much reduced in the hemispheres of lobule VII. It may be noted that in the vermis of lobule VII also ChAT-positive mossy fibers can be observed. Although the origin of specifically these fibers is not known, we have shown that the lateral paragigantocellular nucleus is a likely candidate for similar ChAT-positive mossy fibers to lobule IXa/b of the rat (Jaarsma et al., 1997). Moreover, as the same study shows that the PPTg does not provide mossy fibers to the cerebellar cortex, it was concluded that the ChAT-positive varicose fibers in the cerebellar cortex, at least partly, may be derived from the PPTg. The differential distribution of these ChAT-positive varicose fibers over vermis and hemisphere of lobule VII suggests that the PPTg input may favor vermal areas and, as a consequence, may correlate with a potential STN-PPTg-cerebellar projection (Figure 6).

## Discussion

Recently, in both physiological and pathophysiological conditions, the function of the basal ganglia and the cerebellum systems is increasingly advocated to the subcortical interconnections between these structures (Bostan and Strick, 2010; Helmich et al., 2013; Chen et al., 2014; Louis, 2016; Caligiore et al., 2017). Anatomically, however, disynaptic connections have only been established in primates where they were demonstrated to originate from the cerebellar nuclei to the striatum and from the STN to the cerebellar cortex (for review, see Caligiore et al., 2017). In the present study, we can confirm a disynaptic link from the STN to the cerebellar cortex in rats. However, contrasting primate results we found a more prominent route from the STN to the vermis as compared to the cerebellar hemisphere (see Bostan et al., 2010). Moreover, as anatomical routes from the STN to the pontine nuclei are essentially ill-described, we propose a potential alternative pathway, with the PPTg acting as an intermediary of the disynaptic STN cerebellar pathway.

### The STN-Cerebellar Cortex Link

In our material, we have analyzed nine cases with injections of RABV/CTb into the cerebellar cortex and that had a survival time that did not allow transneuronal labeling beyond 2nd-order labeling. In all these cases, 2nd-order RABV labeling, indeed, was also observed in areas that are known to project to the cerebellar cortex either via the pons (layer V neurons of the cerebral cortex) or via the inferior olive (the prerubral area). This, together with the absence of 3rd-order RABV infected neurons in other layers of the cerebral cortex and within the dorsal thalamus, indicates that the labeling seen in the STN indeed corresponds to 2nd-order RABV infected neurons. Therefore, the conclusion is warranted that STN infection with RABV must have occurred by the synaptic connections of STN terminals to an intermediary that has access to the cerebellar cortex.

In contrast to the results obtained in primates (Bostan et al., 2010), we found a more prominent involvement of the STN after vermal injections as compared to hemispheral injections of the same lobule. In addition, it may be important to stress that we did not observe a clear-cut topographical relation between the location of RABV-infected STN neurons and the location of the RABV/CTb injection site as was also described in the cebus monkey (Bostan et al., 2010). As a whole the STN-cerebellar connection in rodents also seems to be less prominent than that observed in the monkey. However, this assessment should await a more detailed quantitative comparison of numbers of STN numbers relative to size of the injection and its covering of the cerebellar cortex in both rat and monkey.

After confirming the disynaptic STN-cerebellar cortex link in rodents, the intermediary region still remains to be positively identified. Bostan et al. (2010) have proposed that the pontine nuclei (Pn) may serve as the intermediary region (Figure 6, blue route). However, only limited anatomical evidence seems to be available for this proposition (Giolli et al., 2001). Other studies, using diffusion magnetic resonance imaging in healthy volunteers, managed to successfully identify a link between the STN and the Pn and cerebellar cortex (Pelzer et al., 2013; Milardi et al., 2016). The technique used by these studies, however, has limitations (e.g., differentiation between afferents and efferents), and does not provide hard evidence for true axonal fiber pathways (Jbabdi and Johansen-Berg, 2011). Moreover, as there is also no evidence that manifestly links the STN and the Pn in rodents (see e.g., Mihailoff et al., 1989 and Allen Mouse brain connectivity atlas [Internet]), we consider it questionable that the Pn act as an intermediary region in the STN-cerebellar cortex link.

Therefore, as an alternative, we propose the PPTg as the intermediary region in the STN-cerebellar cortex link. The PPTg is a nucleus that not only has been demonstrated to provide cholinergic projections the cerebellar cortex (Jaarsma et al., 1997), but also has been described to maintain reciprocal connections with the STN in the rat and cat (Saper and Loewy, 1982; Edley and Graybiel, 1983). Indeed, our results show beaded ChAT-immunoreactive fibers in the vermis, and these are more sparsely distributed in the paravermis and hemisphere of cerebellar lobule VII, which suggests that the cholinergic projections from the PPTg (Jaarsma et al., 1997) may more prominently reach the vermis of lobule VII as compared to the hemispheres. As such this would correspond nicely with the RABV results that indicate that in the rat it is mainly the vermis of lobule VII that receives the disynaptic input from the STN. Although in our analyzed material, RABV-infected neurons were observed within the PPTg (not shown) only weak and sparse CTb labeling was seen in the PPTg. Possibly, this has to do with the strongly diluted concentration of CTb (1/5 of the normal concentration) that was used in our experiments (see “Materials and Methods” Section) in combination with the thin punctate terminal plexus of ChAT-positive fibers in the molecular layer.

### Functional Considerations of a Potential Cholinergic STN-Cerebellar Link

In order to evaluate the potential role of an STN-PPTg-cerebellar connection in rodents, the following three questions were raised: (1) what, in general, is the role of the STN within basal ganglia functioning; (2) what would be the effect on cholinergic innervation of the cerebellum; and (3) in which behavior does the STN possibly influence the cerebellum?

1. From the anatomy, it is clear that the STN, as an important part of the basal ganglia, plays a role in influencing motor and non-motor behavior (i.e., cognitive and limbic functions). It receives afferent input from the dorsal and ventral pallidum, the cerebral cortex (prefrontal cortex, the first motor and primary somatosensory areas, and the granular insular territory) and the parafascicular nucleus of the thalamus. Its efferents are excitatory and mainly reach the main output stations of the basal ganglia: the globus pallidus interna (GPi) and reticular part of the substantia nigra (Dudman and Gerfen, 2015). This enables the STN, after processing the input it receives, to inhibit behavior by increasing the inhibitory activity of the GPi. As such, the STN lies centrally in the basal ganglia system and may play a pivotal role in both motor and non-motor behavior.
2. If the PPTg is a candidate for acting as an intermediary in the STN-cerebellar cortex link, then it may be hypothesized that increased activity of the STN, not only would lead to an increase of basal ganglia output, but also may affect cerebellar processing as both muscarinic and nicotinic receptors are present in the cerebellar cortex (for review, see Jaarsma et al., 1997). As the PPTg is generally thought to be involved in complex and potentially diverse functions (Gut and Winn, 2016; Takakusaki et al., 2016), it is not simple to establish a functional role of a potential STN-PPTg-cerebellar route. Yet, in this respect it is interesting to mention a recent study using whole-cell patch clamp recordings of rat cerebellar slices that showed that activated muscarinic receptor signaling in the cerebellum can be modulatory by inhibiting long-term potentiation in the cerebellar cortex (Rinaldo and Hansel, 2013). As such, the STN would have the potential to modulate cerebellar learning processes by way of the PPTg.
3. Finally, we could speculate on the type of behavior (i.e., motor and non-motor behavior) that the STN might control by influencing the cerebellum. In this respect it is interesting to note that recently the vermis of lobule VII in the rat has been suggested to be involved in non-motor tasks, as it receives a major disynaptic input from the retrosplenial and orbitofrontal cortices (Suzuki et al., 2012). The paravermis and hemispheric parts of the cerebellar cortex, on the other hand, predominantly receive input from the sensorimotor cortex (Suzuki et al., 2012). Therefore, again contrasting the conclusion by Bostan et al. (2010), in rat the STN would seem to predominantly influence the cerebellum in especially non-motor related behavior.

## Conclusion

Using transneuronal and conventional tracers, we show that a principal basal ganglia nucleus, the STN, is disynaptically connected to the vermis of lobule VII of the cerebellar cortex of the rat. Additionally, we show that the PPTg might be a potential candidate as the intermediary element in this connection. We furthermore suggest that this connection has the potential to modulate cerebellar cortical functioning, especially in non-motor related behavior. It is clear that further assessment of the role of the STN-cerebellar link requires a definite establishment of the intermediary in the STN—cerebellar connection. Furthermore, the observed differences between rat and primates need to be confirmed and further investigated. This information may be vital for understanding the ultimate potential of this basal ganglia—cerebellar connection as well as of its potential role in the generation of pathophysiology of both systems.

## Author Contributions

TJHR and PC designed the experiment. SJ and TJHR analyzed the data and wrote the manuscript.

## Funding

This research was supported by the Dutch Ministry of Health, Welfare and Sports (TJHR and SJ) and the Centre National de la Recherche Scientifique (CNRS) and Aix-Marseille Université through UMR 7289 (PC).

## Conflict of Interest Statement

The authors declare that the research was conducted in the absence of any commercial or financial relationships that could be construed as a potential conflict of interest.
